# Effect of UV Irradiation of Pre-Gel Solutions on the Formation of Collagen Gel Tubes

**DOI:** 10.3390/gels9060458

**Published:** 2023-06-02

**Authors:** Yu Ishibashi, Ryota Haraguchi, Shigehisa Aoki, Yushi Oishi, Takayuki Narita

**Affiliations:** 1 Department of Chemistry and Applied Chemistry, Saga University, Saga 840–8502, Japan; 2 Department of Pathology and Microbiology, Saga University, Saga 849-8501, Japan; aokis@cc.saga-u.ac.jp

**Keywords:** collagen, hollow structure, ultraviolet light modification, photo-cross-linking, pre-gel solution

## Abstract

Hollow collagen gels are promising materials for drug/cell delivery systems to promote tissue regeneration because they may be able to function as carriers for these types of loads. Controlling the cavity size and swelling suppression is essential to expand the applications and improve the usability of such gel-like systems. We investigated the effects of UV-treated collagen solutions as a pre-gel aqueous mixture on the formation and properties of the hollow collagen gels in terms of their preparation range limits, morphology, and swelling ratio. The UV treatment thickened the pre-gel solutions, which allowed hollowing at lower collagen concentrations. This treatment also prevents the over-swelling of the hollow collagen rods in PBS buffer solutions. The UV-treated collagen solutions provided a large lumen space in the prepared collagen hollow fiber rods with a limited swelling ratio, allowing vascular endothelial cells and ectodermal cells to be cultured separately in the outer and inner lumen.

## 1. Introduction

Collagen, a significant extracellular matrix (ECM) component, has been widely studied for tissue engineering applications due to its biocompatibility [1,2,3] and regulatory roles in cell morphology and functions [4,5,6]. Different forms of collagen have been prepared to meet the specific requirements for their use. Sheet-like collagens have been mainly used in medical burn dressings [7,8] and dermal wound healing materials [8,9,10]. Spongy [11,12] and honeycomb-shaped [13,14] scaffolds are necessary for developing three-dimensional cellular tissues. Fiber forms help repair damaged tendons and ligaments due to their anisotropic tensile mechanical properties and filament similarity to natural tissues [15]. High-density collagen xerogel threads have been reported to have the remarkable potential to prevent fibrosis and tissue adhesion due to tissue inflammation [16]. Collagen also has been widely recognized as suitable for delivering various drugs, including growth factors [17,18] and genes [19,20], due to its biodegradability and biocompatibility. Porous collagen materials such as sponge and honeycomb-shaped structures are not only excellent cell scaffolds but can also be utilized as carriers of drugs and cells because the pores inside the collagen could be packed with cells [21,22], nerve growth factor [23], and drugs [21,24,25]. Tubular collagen carriers containing fibrin [26] and heparin [27] have been developed as nerve regeneration-promoting materials. High-density collagen tubes have useful in improving endothelial cell adhesion, and their growth is a potentially promising alternative to vein grafts [28]. We have found a one-pot method to obtain collagen tube gels [29] inspired by the multiple micropores formed by collagen gels prepared by anisotropic buffer diffusion [30,31]. These collagen tubes prepared by our method have a diameter of less than 1 mm. They are expected to contribute significantly to building microvascular networks in regenerative medicine and cultured meat as cell culture carriers and scaffolds. The dried collagen hollow fiber prepared by this method has a Young’s modulus and maximum stress equivalent to those of nylon fibers in gas atmospheres and is easy to handle as a thread [29].

However, water molecules swell the hollow fibers well in an aqueous solution such as PBS, making them too soft and difficult to handle for use as threads or cell carriers. The swelling would indirectly make them significantly more brittle and easier to tear. To avoid unnecessary high swelling and improve the handling of collagen materials in aqueous environments, glutaraldehyde has been widely used as a cross-linking agent, but its carcinogenicity has been reported [32,33,34]. As a non-cytotoxic cross-linking method, a combination of riboflavin (RF) addition and UV-light irradiation has been well-reported for cross-linking of collagen gels [35,36] and corneal collagen [37,38]. RF works as a photosensitizer and triggers the formation of oxygen radicals, which induce chemical bonding between collagen fibers to cross-link [39,40]. In this paper, we investigate the potential of UV treatment using RF to control the desired size of hollow collagen gels and the swelling of their dried products; this study, however, deals with no mechanical strength.

The effect of UV treatment on the rheological properties of the pre-gel solution, such as viscosity, would have considerable impacts on spontaneous pore formation, resulting in shifts in its formable regions and morphologies. In this study, we first evaluated the effect of UV treatment on the viscosity and elasticity of the collagen pre-gel solution using a dynamic rheometer. Then, the morphology of hollow collagen gels prepared by the one-pot method using the UV-treated pre-gel solution was analyzed by microscopy to determine the regions where the tubular shape could be formed. The swelling ratio of the dried collagen tubes immersed in PBS buffer solution was evaluated as an indication of the handling ease.

The hollow collagen gels obtained by the one-pot method should be very interesting for fabricating a novel 3D co-culture system. Co-culture systems can reconstruct more biomimetic environments than monoculture systems [41,42,43], such as promoting tissue formation through direct or indirect cellular interactions [44]. Micropatterned co-culture systems have been reported to localize and coexist with multiple cells in optimal spaces [45], thereby controlling the level of cell-cell interactions [46]. Typically, this co-culture is constructed on a two-dimensional (2D) plane. However, natural tissues or organs have 3D structures; therefore, co-culture systems using 3D scaffold materials are desired for tissue engineering applications that use scaffolds to form the natural structure of interconnected cells. Here, as a model for blood vessels, we attempted to build a 3D co-culture system with vascular endothelial cells inside the hollow collagen scaffold and ectodermal cell tissues on the outside.

## 2. Results and Discussion

### 2.1. Influence of UV Treatment on the Rheological Properties of Collagen Pre-Gel Solutions

**Figure 1:**
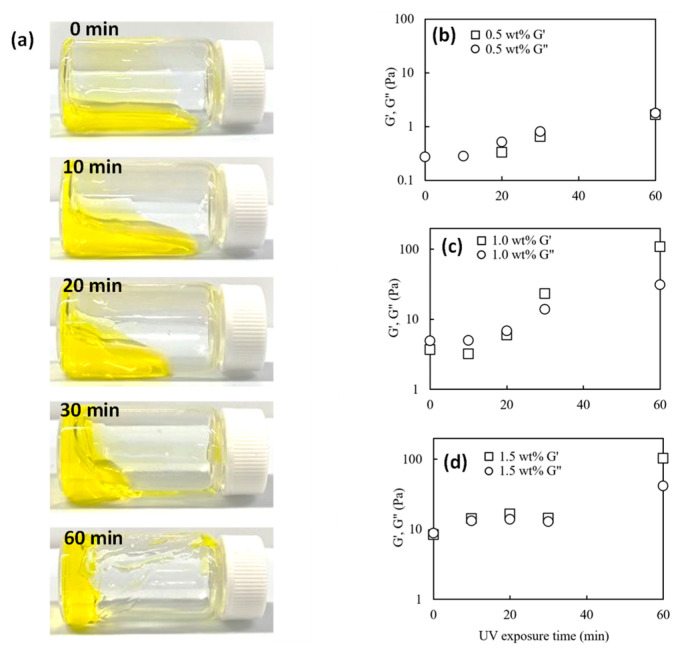
(**a**) Images captured 1 min after the vial containing 1 wt% collagen solution irradiated under the UV light was tilted and laid horizontally at room temperature. These solutions were irradiated for 0, 10, 20, 30, and 60 min; (**b**–**d**) storage modulus G′ and loss modulus G″ at 1 Hz of (**b**) 0.5 wt%, (**c**) 1.0 wt%, and (**d**) 1.5 wt% of the collagen solutions irradiated under UV light for various periods.

Figure 1a displays snapshots showing changes in the deformation properties of the 1 wt% of collagen solutions exposed to UV light for periods of 0, 10, 20, 30, and 60 min and Figure 1b–d graphs showing viscoelastic changes at different concentrations; the collagen solutions presented in this paper all contain 0.02 mM riboflavin. For the 1.0 wt% collagen solutions, the non-UV-treated collagen solution was liquid and deformed its shape as soon as the vial was laid down. In contrast, the UV-treated collagen solution irradiated for 20 min took 1 min to deform and stop flowing. The samples UV-treated for more than 60 min held their shape and failed to flow even after standing for 1 h. The loss and storage modulus increased with UV dose for all collagen solutions, as seen in Figure 1b–d. For the 1.0 and 1.5 wt% collagen solutions, G′ exceeded G″ values over a given UV irradiation time. Since this crossover point (where G′ = G″) represents the beginning of gelation, additional periods of UV irradiation allow collagen solutions to change from liquid to gel. Thus, the 1.0 and 1.5 wt% collagen solutions took 30 and 10 min to gelation, respectively. 0.5 wt% collagen solution had no crossover, whereas G′ and G″ values approached with increasing UV treatment time. UV treatments within 60 min made the 0.5 wt% collagen solution more viscous but did not reach in gel phase. The increase in G′ and G″ values following UV irradiation could be due to the increase in molecular weight resulting from photo cross-linking [45,47].

### 2.2. Effect of UV Irradiation on Cavity Formation Conditions

**Figure 2:**
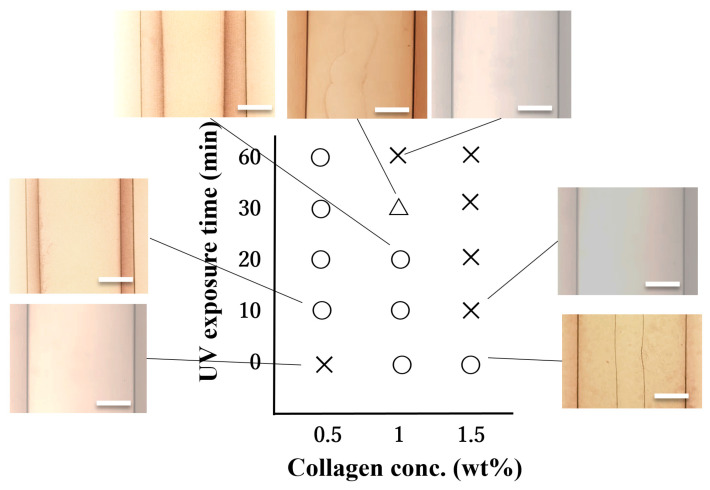
Morphological phase diagram and the typical snapshots of the collagen gel rods as a function of collagen concentration and UV exposure time illustrating the regions where intragel cavities are formed together with their typical shape: long single hollow (**○**), short single hollow (△), and no cavities (×) are observed in the collagen gel rod (scale bars on the images are 0.5 mm).

UV irradiation of aqueous collagen solutions differently altered the hollowing of collagen gel rods. Here we focused on the morphology of collagen rods prepared using UV-treated aqueous collagen solutions as the pre-gel solution (see Figure S1) and the regions in which cavities are formed. Figure 2 shows the phase diagram and the typical images associated with the given regions of the morphology of the tubes designed from various concentrations of collagen solutions exposed and not exposed to UV radiation. This phase diagram distinguishes regions where single and no macroscopic cavities were formed. In the case of the 0.5 wt% collagen pre-gel solution, no cavities were created in the gel prepared with the non-UV-treated solution. The over 10 min UV-treated pre-gel solution generates cavities in the gel rod. In the case of the 1.0 wt% collagen pre-gel solution, hollowing gel rods were produced from the non-UV-exposed solution, but the 30 min treated pre-gel solution resulted in half-hollowing gels, and the cavities were eliminated in the gel rods when using the 60 min UV-treated collagen solution. In the case of the 1.5 wt% collagen pre-gel solution, a single cavity was observed in the gel rod prepared from the unexposed UV solution, while no cavities were formed in the rod designed from the solution exposed to UV for more than 10 min. It is worth mentioning that UV irradiation enabled the formation of hollow collagen gels with a single cavity, even using a low concentration of 0.5 wt% aqueous collagen.

In understanding these cavity formation conditions, comparisons between the dynamic viscoelasticity measurement results (Figure 1) and the cavity formation conditions (Figure 2) would be meaningful. Comparing G′ and G″ values and pore formation conditions indicates that no specific viscosity or elasticity values are required for pore formation. Cavities in gel rods were formed in liquid state pre-gels close to the gel state where G′ and G″ cross over.

The effects of UV treatment will be considered below, including the principle of single pore formation. The formation of a single pore follows almost the same principle as that of collagen gels with cylindrical holes aligned parallel to the collagen growth direction [30,48], which is obtained when collagen acidic solution is neutralized by contacting it with neutral buffer solutions. The holes are formed by phase separation, simultaneously forming a three-dimensional gel network. In our Teflon tube, the buffer solution diffusion as a gelling environment induces a morphology by phase in conjunction with a pinning effect, the entire process of macrophase separation being directed from the top to the bottom end of the tube vertically oriented. The gelated region contracts due to the charge cancelation of the collagen (as a result of the close values of the diffusing environment pH and the isoelectric point of collagen), leading to a space consisting of the aqueous phase close to the Teflon tube wall [29]. The gelation buffer solution penetrating through this crack leads to gelling and confining the collagen along the wall, lowering the collagen concentration at the center of the Teflon tube to form an aqueous phase. This aqueous phase will become a single pore. From previous results, lower concentrations of collagen solution tend to undergo more quickly such a macroscopic phase separation, thus producing a macroscopic pore [29,31]. Non-UV-treated 0.5 wt% collagen solutions may fail to form complete cavities because the concentrated collagen wall is too thin. In contrast, the UV-irradiated 0.5 wt% collagen solution could form thin gel tubes even at low concentrations. The thickening by UV irradiation could pin the phase separation in the early process and prevent the collagen-concentrated tube walls from collapsing. The 1.5 wt% collagen solution forms a gel with UV treatment before interacting with the buffer solution, as shown in Figure 1. Collagen in the gel state has higher elasticity than in the sol state, and we assume that pores were not formed.

### 2.3. Cavity Ratio of the Single Pore in Collagen Gel Rod

**Figure 3:**
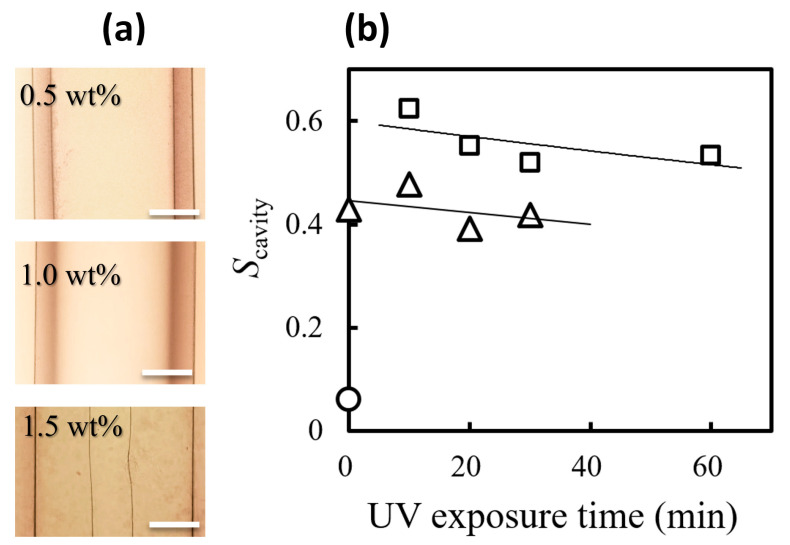
The cavity of collagen gel rods: (**a**) typical images of the cavities inside collagen gel rods prepared with 0.5 wt% and 1.0 wt% collagen solutions treated under UV light for 10 min, and 1.5 wt% solutions treated with non-UV irradiation (from top to bottom); (**b**) cavity ratio *S*_cavity_ as a function of the UV exposure time to the pre-gel solutions of different concentrations (scale bars on the images are 0.5 mm).

The microscopic images in Figure 3a suggest that the wall thickness of the formed gels increases with the collagen concentration used; the cavity size in the collagen gel rods is related mainly to the pre-gel solution. The cavity ratio (*S*_cavity_ = *d*_i_^2^/*d*_o_^2^) can characterize the size of the cavity inside the gel. The cavity ratio decreased with distance from the diffusion end but reached an equilibrium value over a certain length. Figure 3b shows the effect of UV exposure time on the equilibrium cavity ratio *S*_cavity_. The cavity ratio *S*_cavity_ increased with decreasing collagen concentration, in agreement with previous reports [29]. For the sensitivity to UV irradiation at the same collagen concentration, *S*_cavity_ showed slightly lower values in longer UV irradiation time. Since the cavities are formed by phase separation [30,48], the hollowing could be de-accelerated by gelation or thicking before they are well-separated and develop a mature tube. UV irradiation makes the collagen solution viscous, and then the thickened collagen solution may freeze phase separation at the early stage, which could prevent cavity growth. The cavity ratio in the collagen gel obtained with the 0.5 wt% collagen solution is about 0.2 higher than that obtained with the 1 wt% solution, as shown in Figure 3b. This result demonstrates that UV treatment applied to low-concentration aqueous collagen solutions can thicken them and allow them to form a wider single cavity. The range of *S*_cavity_ values in our study was 0.08–0.6 even for 1 wt% collagen gel rods; this value is much larger than that reported in the previous work (*S*_cavity_ = 0.02–0.2) [29]. These substantially increased *S*_cavity_ values in this work may be due to the influence of the addition of riboflavin or the use of Teflon tubing as the preparation template.

### 2.4. The Swelling Ratio of Collagen Gel Tubes Prepared by UV-Treated Pre-Gel Solution

**Figure 4:**
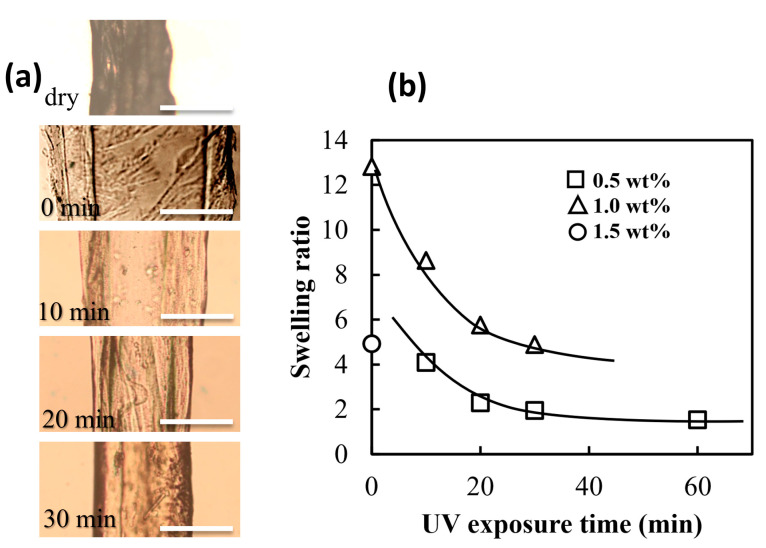
Swelling of dried collagen gel rods after immersed in PBS buffer solution for one day: (**a**) snapshots of the dried then re-swollen collagen gel rods obtained from 0.5 wt% collagen solutions treated under given UV irradiation periods; (**b**) swelling ratio (*d*_so_ − *d*_si_)^2^/(*d*_do_ − *d*_di_)^2^ of the re-swollen collagen gels as a function of collagen concentration and the UV exposure time. The scale bars on the images are 0.5 mm.

The collagen gel tubes prepared with non-treated UV collagen solution swell well in PBS buffer solution and are so brittle that they have been impossible to handle with tweezers and, thus, practically unusable in aqueous solutions. As an indicator of the difficulty of handling the wetted collagen tubes, the swelling ratio of hollow collagen gels in PBS buffer solution was examined here. The swelling ratio was calculated as (*d*_so_ − *d*_si_)^2^/(*d*_do_ − *d*_di_)^2^, where *d*_so_, *d*_si_, *d*_do_, and *d*_di_ were the outer and inner diameters of swollen and dried collagens, respectively. Figure 4 shows the dependence of the UV exposure time of the pre-gel collagen solution on the re-swelling of the dried gel rods; the collagen rods dried in an incubator for 1 day at 20 °C were immersed in PBS buffer solution for 1 day to obtain re-swollen collagen rods. The re-swollen rods prepared from longer-time UV-exposed collagen solutions became smaller in the swelling ratio. One reason for the reduction in the swelling ratio would be the insolubilization caused by the increase in the collagen molecular weight [49,50] in the aqueous solution due to UV irradiation; the idea that UV irradiation increases the molecular weight of collagen was based indirectly on the fact that UV irradiation causes the collagen solution to thicken. As the collagen becomes a longer chain, the dissolution entropy decreases, thus lowering the solubility of the collagen molecules in the PBS solution. A longer time of UV exposure leads to chemical cross-linking throughout the pre-gel solution of collagen, resulting in a substantial decrease of cross-linked collagen solubility in aqueous media. While the contact angles of the collagen films obtained by drying the UV-treated collagen solution decreased as the UV irradiation time increased (see Figure S2). The results of contact angle measurement indicate that the affinity of collagen for water is reduced, but this is not evidence that UV treatment increases the molecular weight of collagen. Hence, the decrease in the swelling ratio with UV treatment could be not only due to the increased molecular weight of the collagen molecule but also to the chemically induced hydrophobization of the collagen molecule [51,52].

The dependence of the collagen concentration on the swelling ratio appeared inconsistent; higher swelling ratios were observed for collagen rods prepared at 1, 0.5, and 1.5 wt%, in that order. Ascending values of the equilibrium swelling ratio experimentally obtained with increasing concentrations of collagen solutions could be explained by a progressively larger amount of PBS solution required to reach equilibrium swelling of a larger and larger amount of polymer per volume unit. The unexpectedly lower swelling ratio observed in the rod formed by 1.5 wt% collagen solution could be due to insufficient water loss during the drying process performed one day.

### 2.5. Usefulness as a Co-Culture Scaffold

**Figure 5:**
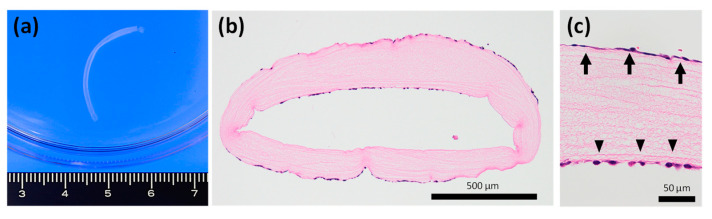
Collagen tube as a cell culture scaffold: (**a**) macroscopic image of collagen tube in PBS buffer solution; (**b**) keratinocyte and endothelial cells cocultured by the collagen tube. Representative sections stained with hematoxylin-eosin (HE); (**c**) Arrows indicate keratinocytes, and arrowheads indicate endothelial cells.

In this study, we developed a small-diameter collagen tube optimized as a cell culture scaffold. The tube had a lumen diameter of approximately 0.5 mm and a cavity ratio *S*_cavity_ of 0.64 when hydrated. As a result of cell culture design, Keratinocytes, and vascular endothelial cells adhered and proliferated onto the outer surface and inner lumen of the collagen tube, respectively (Figure 5). Although there are few reports on the co-culture of different cell types using hollow collagen fibers, our collagen tube displayed potential as a cell scaffold and much ease of handling because of the lower swelling ratio. This allows the seeding and co-culturing of different types of cells on the outer surface and inner lumen of the tube without special methods. Our results suggest the possibility of a breakthrough material that can provide vascular networks and nerve fibers in artificial organ fabrication and regenerative medicine technology, although further investigations are required to determine whether it has excellent biocompatibility and cell growth properties.

## 3. Conclusions

We have demonstrated that UV-treated collagen pre-gel solutions are available to prepare collagen tubular gels with significant hollowness and can prevent collapse even in solution. The tubular gels could be prepared by filling Teflon tubes with the collagen solution and gelating it with carbonate buffer solution from one end of the Teflon tube. Viscoelastic behavior of the studied collagen-based systems exhibited an increasing tendency of both viscous and elastic components at UV irradiation time and collagen concentration, with a prevalence of elastic behavior at UV irradiation times above a certain threshold value depending on the collagen concentration. The hollow gels could be developed when the pre-gel solution is in a liquid state close to the gel state. Lower concentration collagen solution allowed the preparation of hollow gels with thinner walls. UV treatment reduced the swelling ratio of dried tubes in PBS buffer solution from about 13 to less than 1.6. Collagen tubes with wide open pore sizes were valuable as carriers for co-cultivation. The collagen tubes with wide pore size could be used not only as scaffolds for co-culture cell culture carriers but also as multi-carriers for drug delivery systems; thus, these findings could be very promising approaches for practical applications of such materials in regenerative medicine for the construction of vascular and neural networks, and in non-animal drug models.

## 4. Materials and Methods

### 4.1. Materials

Collagen (type-I atelo-collagen) was acquired from Nippon Ham Foods Co., Ltd., Osaka, Japan. Carbonate buffer was purchased from DKK-TOA Co., Ltd., Tokyo, Japan, as a pH 10.02 standard solution powder reagent, 0.1 M Hydrochloric Acid, and riboflavin from Wako Pure Chemical Industries (Osaka, Japan). These chemicals were used without further purification.

### 4.2. Preparation of Collagen Pre-Gel Solution and Its UV-Light Irradiation

A primary collagen solution was freeze-dried, and the obtained powder was dissolved in pH 3 HCl solution to obtain 0.5, 1.0, and 1.5 wt% collagen solutions. The pH 3 HCl solution was obtained by diluting 0.1 M HCl solution with distilled water. Carbonate buffer as a gelling environment was prepared by diluting the powdered reagent with distilled water to 25 mM. These collagen solutions and riboflavin were mixed in a beaker and stirred with a magnetic stirrer in a refrigerator (7 °C) for about 12 h to obtain 0.02 mM riboflavin-mixed collagen solutions. The riboflavin-mixed collagen solutions were irradiated with a UV-light source (UV Lamp 4, 8 W, Camag, Muttenz, Switzerland) at a wavelength of 366 nm for 10, 20, 30, and 60 min from 15 cm above the bottom of the beaker at room temperature.

### 4.3. Preparation of Collagen Hydrogel Rods

The UV-treated collagen solutions were filled inside a PTFE tube (NICHIAS Corp., Tokyo, Japan, NAFLON PTFE tube, 1.59 mm inner diameter, 35 mm long) and sealed at one end with a fluorine sealing tape. The PTFE tube containing the collagen solutions was immersed vertically in a disposable optical cell (10 mm × 10 mm × 45 mm) filled with 3 mL of the carbonate buffer solution; the sealed end of the PTFE tube was placed down, and the opposite end of the tube was covered with the buffer solution. The disposable optical cell was refrigerated (7 °C) for 5 days. For comparison, we also prepared samples gelated in 0.02 mM riboflavin-mixed collagen solution without UV exposure. We have placed the drawing illustrating this collagen hollow gel preparation method in Figure S1.

### 4.4. Dynamic Viscoelasticity Measurement

Dynamic viscoelasticity measurements were performed at a controlled temperature of 25 °C on the pregel solutions subjected to 0, 10, 20, 30, and 60 min of UV treatment (Figure S2). The storage and loss moduli were determined using a rotary rheometer MCR-101 (Anton Paar, Graz, Austria) with plate-plate geometry (25 mm, 1 mm gap). Measurements were performed in the frequency (ω) range from 0.1 rad/s to 10 rad/s with a strain value of 0.1%.

### 4.5. Microscopic Observation and Analysis

The collagen gel rods were observed under brightfield conditions with an optical microscope (Leica DMI3000 B, Leica Microsystems, Wetzlar, Germany) at room temperature. The microscopy images of the gels were captured using a digital camera (QICAM Fast 1394; QIMAGING, Burnaby, BC, Canada). The hollow cavity diameter inside the gel (*d*_i_) and the outer diameter of the gel rod (*d*_o_) were obtained from the optical microscopy images captured 20 mm away from the PTFE tube end directly in contact with the carbonate buffer (diffusion end). The cavity area per cross-sectional area was calculated as *d*_i_^2^/*d*_o_^2^.

### 4.6. Swelling Ratio Measurement

After the gel rods were removed from the PTFE tube by pushing them out with a dropper, the extracted rods were pinched at one end, hung, and dried in a constant temperature and humidity chamber at 20 °C and 60% RH for 24 h. The dried collagen gel rods were immersed in PBS (pH 7) for 24 h at room temperature to re-swell the gel rods, and the diameter of the dried rods (*d*_d_) and the diameter of the re-swollen collagen rods (*d*_s_) were determined by optical microscopy as described in 4.4. The swelling ratio was calculated as (*d*_so_ − *d*_si_)^2^/(*d*_do_ − *d*_di_)^2^, where *d*_so_, *d*_si_, *d*_do_, and *d*_di_ were the outer and inner diameters of swollen and dried collagens, respectively.

### 4.7. Cell Culture and Observation

Mouse endothelial cell line MS-1 (CRL-2279) was obtained from the ATCC (Rockville, MD, USA). Human keratinocyte cell line HaCaT was obtained from CLS Cell Lines Service GmbH (Köln, Germany). Cells were cultured in RPMI-1640 medium (Fujifilm, Tokyo, Japan) containing 10% fetal bovine serum (Nichirei Biosciences, Tokyo, Japan), 100 μg/mL streptomycin, and 100 μg/mL penicillin. The culture medium was changed every 2 days. For co-culture experiments, 5 × 10^5^ endothelial cells were injected into the lumen of the collagen tube, followed by seeding 5 × 10^5^ keratinocytes on the outer surface of the tube. Samples were fixed with 10% formalin and embedded in paraffin after 5 days of culture. Deparaffinized sections were stained with hematoxylin-eosin (HE).

## Data Availability

Not applicable.

## Supplementary Materials

The following supporting information can be downloaded at: https://www.mdpi.com/article/10.3390/gels9060458/s1, Figure S1: Schematic diagram illustrating the preparation method of thin hollow gels from UV-treated collagen solution. Figure S2: Contact angles of sheets dried after casting UV-irradiated collagen solution.

## References

[1] Glowacki J., Mizuno S. (2008). Collagen scaffolds for tissue engineering. Biopolym. Orig. Res. Biomol..

[2] Dong C., Lv Y. (2016). Application of collagen scaffold in tissue engineering: Recent advances and new perspectives. Polymers.

[3] Takezawa T. (2003). A strategy for the development of tissue engineering scaffolds that regulate cell behavior. Biomaterials.

[4] Kyriakopoulou K., Piperigkou Z., Tzaferi K., Karamanos N.K. (2023). Trends in extracellular matrix biology. Mol. Biol. Rep..

[5] Elosegui-Artola A. (2021). The extracellular matrix viscoelasticity as a regulator of cell and tissue dynamics. Curr. Opin. Cell Biol..

[6] Kirkness M.W.H., Lehmann K., Forde N.R. (2019). Mechanics and structural stability of the collagen triple helix. Curr. Opin. Chem. Biol..

[7] Wang Y., Beekman J., Hew J., Jackson S., Issler-Fisher A.C., Parungao R., Lajevardi S.S., Li Z., Maitz P.K.M. (2018). Burn injury: Challenges and advances in burn wound healing, infection, pain and scarring. Adv. Drug Deliv. Rev..

[8] Peng W., Li D., Dai K., Wang Y., Song P., Li H., Tang P., Zhang Z., Li Z., Zhou Y. (2022). Recent progress of collagen, chitosan, alginate and other hydrogels in skin repair and wound dressing applications. Int. J. Biol. Macromol..

[9] Ruszczak Z. (2003). Effect of collagen matrices on dermal wound healing. Adv. Drug Deliv. Rev..

[10] Sharma S., Rai V.K., Narang R.K., Markandeywar T.S. (2022). Collagen-based formulations for wound healing: A literature review. Life Sci..

[11] Shiekh P.A., Andrabi S.M., Singh A., Majumder S., Kumar A. (2021). Designing cryogels through cryostructuring of polymeric matrices for biomedical applications. Eur. Polym. J..

[12] Chen G., Kawazoe N. (2016). Collagen-Based Porous Scaffolds for Tissue Engineering. Biomaterials from Nature for Advanced Devices and Therapies.

[13] George J., Onodera J., Miyata T. (2008). Biodegradable honeycomb collagen scaffold for dermal tissue engineering. J. Biomed. Mater. Res. Part A Off. J. Soc. Biomater. Jpn. Soc. Biomater. Aust. Soc. Biomater. Korean Soc. Biomater..

[14] Itoh H., Aso Y., Furuse M., Noishiki Y., Miyata T. (2001). A honeycomb collagen carrier for cell culture as a tissue engineering scaffold. Artif. Organs.

[15] Cornwell K.G., Lei P., Andreadis S.T., Pins G.D. (2007). Cross-linking of discrete self-assembled collagen threads: Effects on mechanical strength and cell–matrix interactions. J. Biomed. Mater. Res. Part A.

[16] Aoki S., Takezawa T., Nagase K., Oshikata-Mitazaki A., Morito S., Sakumoto T., Masuda M., Yamamoto-Rikitake M., Akutagawa T., Toda S. (2019). A high-density collagen xerogel thread prevents the progression of peritoneal fibrosis. Biomater. Sci..

[17] Hu J., Song Y., Zhang C., Huang W., Chen A., He H., Zhang S., Chen Y., Tu C., Liu J. (2020). Highly aligned electrospun collagen/polycaprolactone surgical sutures with sustained release of growth factors for wound regeneration. ACS Appl. Bio. Mater..

[18] Parenteau-Bareil R., Gauvin R., Berthod F. (2010). Collagen-based biomaterials for tissue engineering applications. Materials.

[19] Curtin C.M., Cunniffe G.M., Lyons F.G., Bessho K., Dickson G.R., Duffy G.P., O’Brien F.J. (2012). Innovative collagen nano-hydroxyapatite scaffolds offer a highly efficient non-viral gene delivery platform for stem cell-mediated bone formation. Adv. Mater..

[20] Dang J.M., Leong K.W. (2006). Natural polymers for gene delivery and tissue engineering. Adv. Drug Deliv. Rev..

[21] Ke T., Yang M., Mao D., Zhu M., Che Y., Kong D., Li C. (2015). Co-transplantation of skin-derived precursors and collagen sponge facilitates diabetic wound healing by promoting local vascular regeneration. Cell. Physiol. Biochem..

[22] Wen P., Wu W., Wang F., Zheng H., Liao Z., Shi J., Zhu C., Zhao P., Cheng H., Li H. (2023). Cell delivery devices for cancer immunotherapy. J. Control. Release.

[23] Han Q., Sun W., Lin H., Zhao W., Gao Y., Zhao Y., Chen B., Xiao Z., Hu W., Li Y. (2009). Linear ordered collagen scaffolds loaded with collagen-binding brain-derived neurotrophic factor improve the recovery of spinal cord injury in rats. Tissue Eng. Part A.

[24] Choudhary S., Sharma K., Sharma V., Kumar V., Sehgal R. (2022). Marine Collagen for Delivery of Therapeutics. Marine Biomaterials: Therapeutic Potential.

[25] Mederle N., Marin S., Marin M.M., Danila E., Mederle O., Albu Kaya M.G., Ghica M.V. (2016). Innovative biomaterials based on collagen-hydroxyapatite and doxycycline for bone regeneration. Adv. Mater. Sci. Eng..

[26] Kuffler D.P., Reyes O., Sosa I.J., Santiago-Figueroa J. (2011). Neurological recovery across a 12-cm-long ulnar nerve gap repaired 3.25 years post trauma: Case report. Neurosurgery.

[27] Long Q., Wu B., Yang Y., Wang S., Shen Y., Bao Q., Xu F. (2021). Nerve guidance conduit promoted peripheral nerve regeneration in rats. Artif. Organs.

[28] Li X., Xu J., Nicolescu C.T., Marinelli J.T., Tien J. (2017). Generation, endothelialization, and microsurgical suture anastomosis of strong 1-mm-diameter collagen tubes. Tissue Eng. Part A.

[29] Iwamoto Y., Haraguchi R., Nakao R., Aoki S., Oishi Y., Narita T. (2022). One-Pot Preparation of Collagen Tubes Using Diffusing Gelation. ACS Omega.

[30] Furusawa K., Sato S., Masumoto J.-i., Hanazaki Y., Maki Y., Dobashi T., Yamamoto T., Fukui A., Sasaki N. (2012). Studies on the formation mechanism and the structure of the anisotropic collagen gel prepared by dialysis-induced anisotropic gelation. Biomacromolecules.

[31] Furusawa K., Mizutani T., Machino H., Yahata S., Fukui A., Sasaki N. (2015). Application of multichannel collagen gels in construction of epithelial lumen-like engineered tissues. ACS Biomater. Sci. Eng..

[32] Zeiger E., Gollapudi B., Spencer P. (2005). Genetic toxicity and carcinogenicity studies of glutaraldehyde—A review. Mutat. Res. /Rev. Mutat. Res..

[33] Takigawa T., Endo Y. (2006). Effects of glutaraldehyde exposure on human health. J. Occup. Health.

[34] Sarrigiannidis S.O., Rey J.M., Dobre O., González-García C., Dalby M.J., Salmeron-Sanchez M. (2021). A tough act to follow: Collagen hydrogel modifications to improve mechanical and growth factor loading capabilities. Mater. Today Bio.

[35] Tirella A., Liberto T., Ahluwalia A. (2012). Riboflavin and collagen: New cross-linking methods to tailor the stiffness of hydrogels. Mater. Lett..

[36] Adamiak K., Sionkowska A. (2020). Current methods of collagen cross-linking. Int. J. Biol. Macromol..

[37] Kohlhaas M., Spoerl E., Schilde T., Unger G., Wittig C., Pillunat L.E. (2006). Biomechanical evidence of the distribution of cross-links in corneastreated with riboflavin and ultraviolet A light. J. Cataract Refract. Surg..

[38] Spoerl E., Huhle M., Seiler T. (1998). Induction of cross-links in corneal tissue. Exp. Eye Res..

[39] Voicescu M., Ionita G., Constantinescu T., Vasilescu M. (2006). The oxidative activity of riboflavin studied by luminescence methods: The effect of cysteine, arginine, lysine and histidine amino acids. Rev. Roum. De Chim..

[40] Raiskup F., Spoerl E. (2013). Corneal cross-linking with riboflavin and ultraviolet AI Principles. Ocul. Surf..

[41] Kook Y.-M., Jeong Y., Lee K., Koh W.-G. (2017). Design of biomimetic cellular scaffolds for co-culture system and their application. J. Tissue Eng..

[42] Mierke C.T. (2023). Physical and biological advances in endothelial cell-based engineered co-culture model systems. Seminars in Cell & Developmental Biology.

[43] Guo L., Zhu Z., Gao C., Chen K., Lu S., Yan H., Liu W., Wang M., Ding Y., Huang L. (2022). Development of Biomimetic Hepatic Lobule-Like Constructs on Silk-Collagen Composite Scaffolds for Liver Tissue Engineering. Front. Bioeng. Biotechnol..

[44] Battiston K.G., Cheung J.W.C., Jain D., Santerre J.P. (2014). Biomaterials in co-culture systems: Towards optimizing tissue integration and cell signaling within scaffolds. Biomaterials.

[45] Tenje M., Cantoni F., Hernández A.M.P., Searle S.S., Johansson S., Barbe L., Antfolk M., Pohlit H. (2020). A practical guide to microfabrication and patterning of hydrogels for biomimetic cell culture scaffolds. Organs-A-Chip.

[46] Lee H.W., Kook Y.-M., Lee H.J., Park H., Koh W.-G. (2014). A three-dimensional co-culture of HepG2 spheroids and fibroblasts using double-layered fibrous scaffolds incorporated with hydrogel micropatterns. RSC Adv..

[47] Li J., Lewis C.L., Chen D.L., Anthamatten M. (2011). Dynamic mechanical behavior of photo-cross-linked shape-memory elastomers. Macromolecules.

[48] Maki Y., Furusawa K., Yamamoto T., Dobashi T. (2018). Structure formation in biopolymer gels induced by diffusion of gelling factors. J. Biorheol..

[49] Kakehashi A., Akiba J., Ueno N., Chakrabarti B. (1993). Evidence for singlet oxygen-induced cross-links and aggregation of collagen. Biochem. Biophys. Res. Commun..

[50] McCall A.S., Kraft S., Edelhauser H.F., Kidder G.W., Lundquist R.R., Bradshaw H.E., Dedeic Z., Dionne M.J.C., Clement E.M., Conrad G.W. (2010). Mechanisms of corneal tissue cross-linking in response to treatment with topical riboflavin and long-wavelength ultraviolet radiation (UVA). Investig. Ophthalmol. Vis. Sci..

[51] Wang W., Zhang Y., Ye R., Ni Y. (2015). Physical crosslinkings of edible collagen casing. Int. J. Biol. Macromol..

[52] Paul R.G., Bailey A.J. (2003). Chemical stabilisation of collagen as a biomimetic. Sci. World J..

