# Supplementary material for: Effect of UV Irradiation of Pre-Gel Solutions on the Formation of Collagen Gel Tubes
Source: Gels. 2023 Jun 2;9(6):458. doi: 10.3390/gels9060458 (PMC10298169; doi:10.3390/gels9060458)
Supplement: Supplementary file 1 [file gels-09-00458-s001.zip › gels-2398374-supplementary.pdf]

Supporting Information

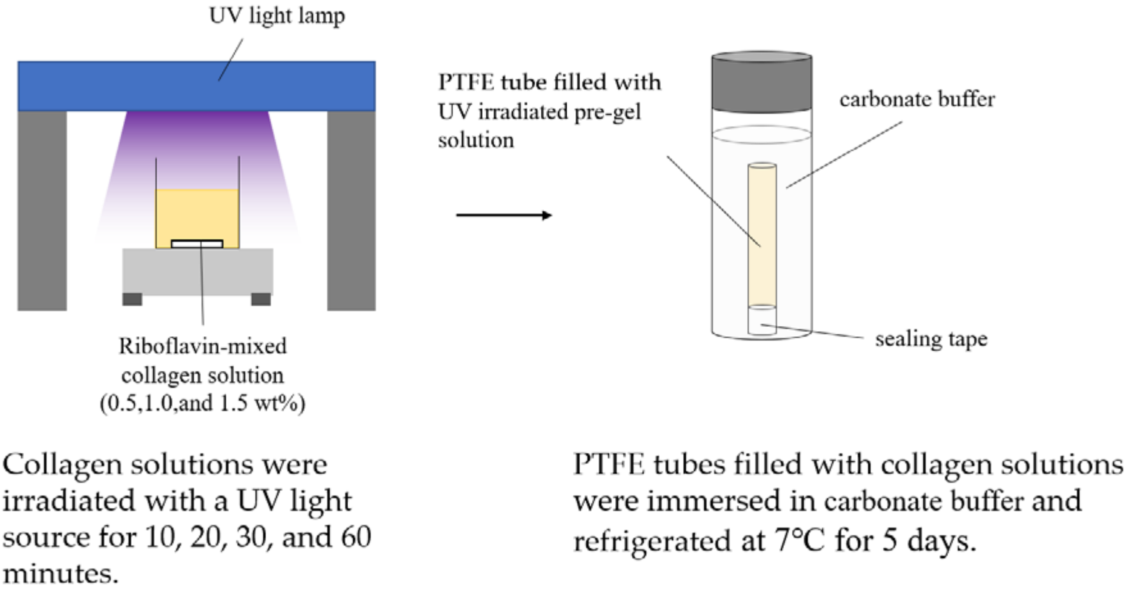

Figure S1. Schematic diagram illustrating the preparation method of thin hollow collagen gel rod from the UV-treated collagen solution.

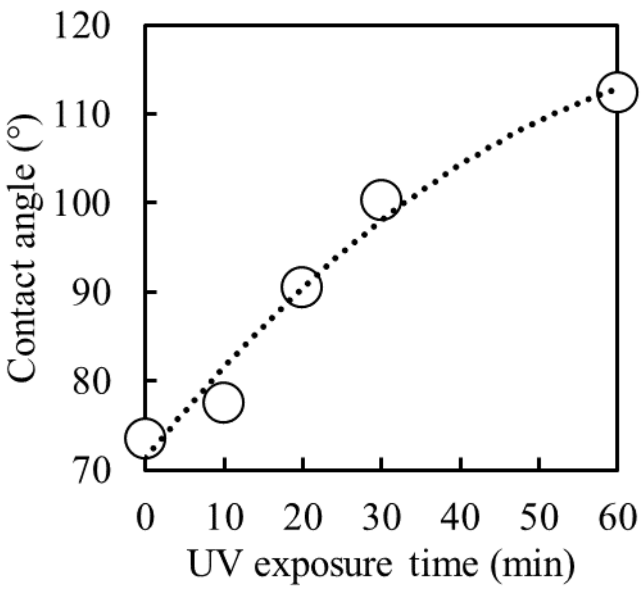

Figure S2. Contact angles of sheets dried after casting UV-irradiated collagen solution: The contact angles between the collagen sheet and the droplet formed by dropping 5 μL of ultrapure water on various collagen sheets were measured at 20 °C. The collagen sheets were obtained by the following method: a drop of 20 μl of the given UV-treated 1 wt% aqueous collagen solution was dropped onto a glass slide, cast, and then dried in a constant temperature and humidity chamber at 20 °C and 60% RH for 24 hours.
